# Deep Learning for Disease Detection: Building a Leaf Image Classifier for Roses

**DOI:** 10.3390/s26103023

**Published:** 2026-05-11

**Authors:** Mihnea Ș. Georgescu, Silviu Răileanu, Camelia Ungureanu, Diana Elena Vizitiu

**Affiliations:** 1 Faculty of Automatic Control and Computer Science, National University of Science and Technology Politehnica Bucharest, 313 Splaiul Independentei, Sector 6, 060042 Bucharest, Romania; mihnea.georgescu@stud.acs.upb.ro; 2 Faculty of Chemical Engineering and Biotechnology, National University of Science and Technology Politehnica Bucharest, 313 Splaiul Independentei, Sector 6, 060042 Bucharest, Romania; camelia.ungureanu@upb.ro; 3 National Research and Development Institute for Biotechnology in Horticulture Stefanesti Arges, Soseaua Bucharest–Pitesti Street, No 37, 117715 Stefanesti, Romania; vizitiud@yahoo.com

**Keywords:** deep learning, leaf disease detection, machine vision, image classification, neural networks, agriculture

## Abstract

**Highlights:**

**What are the main findings?**

- Three deep learning architectures show very high accuracy for automated leaf screening.
- The architecture is insensitive to intensive preprocessing by masking and shows high accuracy on faster workflows.

**What is the implication of the main finding?**

- The use of deep learning is viable for the automation of disease detection for roses in a practical workflow.

**Abstract:**

Early and reliable detection of rose diseases is important for automating plant monitoring and timely intervention throughout the crop lifecycle. In this context, leaf-image analysis combined with machine learning offers a practical approach for disease detection in roses. This study tests a binary classification framework that distinguishes diseased leaves using convolutional neural networks (CNNs). Three architectures were evaluated: a lightweight CNN trained from scratch as a baseline model, and two residual network models fine-tuned through transfer learning from weights pretrained on a large-scale visual recognition dataset. To assess robustness, two preprocessing strategies were also compared: a lightweight hue-based leaf isolation method that preserves full color information, and a grayscale conversion approach without masking. Experimental results obtained on a small held-out test set show strong classification performance across all evaluated models. At the same time, the findings indicate that additional validation is needed on more diverse datasets to confirm generalization under varying lighting conditions, background complexity, and plant growth stages. The results support the feasibility of CNN-based disease detection for roses and highlight its potential for integration into automated monitoring workflows.

## 1. Introduction

The rose is a species of the Rosaceae family, and is one of the most widespread and adapted ornamental flowering shrubs cultivated in urban environments [1]. It is a plant appreciated for its beauty and perfume, but also for its multiple uses in the medicinal and chemical fields. The fruits, petals, and leaves can be added to the composition of certain products, such as cosmetics, foods, and pharmaceutical preparations. From a therapeutic point of view, the rose is associated with beneficial effects in improving inflammation, regulating blood sugar, reducing menstrual pain, combating depression and stress, as well as supporting the health of the nervous system and slowing down the aging process. Rose water is also recognized for its skin care properties, having antibacterial action against various types of microorganisms [2,3,4].

Rose leaf diseases can reduce yield and plant quality. Disease monitoring is traditionally performed visually by specialists, which is inefficient in commercial floriculture [5] because it involves a considerable amount of work, expertise in phytopathology, and extended processing periods [6].

Pathogen-induced diseases represent a significant limitation in rose cultivation, negatively influencing the ornamental value of the plants and the integrity of the gardens. Under protected conditions, these diseases compromise the quality of cut flowers and can generate significant economic losses. Even minor attacks on the foliage or petals can have disproportionate effects. Fungi are the main pathogens and are responsible for a wide range of diseases in various rose-growing areas [7].

Powdery mildew of roses, caused by the pathogen *Podosphaera pannosa*, is an important pathogen that causes a decrease in crop yield and quality by colonizing mycelium on various plant organs, negatively influencing their commercial value. This pathology is widespread in rose-growing areas, both in protected environments and in open crops [8].

Rose crops are vulnerable to fungal infections, such as powdery mildew and black spot [7]. Visual inspection is slow, subjective, and difficult to standardize across people and time, while delayed detection increases treatment costs and crop loss. The goal of this work is to automate early-stage screening using supervised deep learning on leaf images, producing a simple outcome that can be integrated into a practical workflow.

This is a binary classification task, distinguishing between healthy leaves and diseased leaves. Supervised learning is applied to train the model on examples of images paired with their corresponding labels, thereby establishing a mapping from pixel values to a class prediction. Convolutional neural networks (CNNs) were selected as the model family for this application because CNNs can identify local patterns (i.e., filters) irrespective of their position in an image, making them suitable for images.

There are two particular areas of importance in this context. The first area is related to the fact that the data were generated by the authors themselves; therefore, the authors apply data augmentation and transfer learning to increase sample efficiency and decrease the possibility of overfitting. The second area is related to the fact that the cost associated with misclassifying diseased leaves (false negatives) and misclassifying healthy leaves (false positives) is often asymmetric. Therefore, this study includes accuracy, but also discusses why other metrics and more comprehensive, diverse data for evaluation will need to be included in order to demonstrate deployment readiness.

The early plant disease classifiers relied on handcrafted color and texture descriptors [9] as inputs into shallow models [10]; however, these systems suffered greatly from degradation due to changing lighting, pose, and background clutter of the leaf. Modern systems have significantly improved upon the performance of the early systems by utilizing CNNs that were pretrained on large-scale natural-image datasets (e.g., ImageNet) [11] and fine-tuned on agricultural imagery, resulting in significant improvements in robustness and the ability to perform with less labeled data specific to crops. A particularly popular dataset for promoting this paradigm is the PlantVillage [12] dataset, which has allowed for cross-crop transfer. It has been reported that deep models can achieve accuracy greater than 99% across multiple diseases when there is enough labeled data available, although the numbers are often dependent on the conditions of the dataset and the evaluation protocols [13].

A number of architectural trends have emerged in relation to the development of deep and trainable models. One of the most important was the introduction of residual networks (ResNets) [14], which include skip connections that enable gradients to flow through many layers, allowing for deeper models to be trained without suffering from the same optimization challenges faced by the previous CNNs. Another recent family of models, EfficientNet [15,16], emphasizes accuracy–parameter trade-offs, which is important for deploying the model on limited hardware.

Systems-based approaches to real-world deployments reveal that many failures do not occur because the model itself is theoretically capable, but rather because the data distribution changes. Changes in lighting, camera angle, new cultivars, seasonality, different disease stages, and changes in the backgrounds of the input images can result in a changed representation of the images relative to the training set [17]. As a result, thorough validation (test set held out, repeated splits, and ideally external test sets) is needed, and motivates lightweight preprocessing that minimizes irrelevant variation [18]. In this project, the use of leaf masking is considered as a pragmatic means of minimizing dependency on background in a small-data regime—it is not intended to be a full segmentation or diagnostic solution.

In practice, pretraining on large, general image datasets enables the model to learn reusable visual features (e.g., edges, textures, and simple shapes). Then, fine-tuning is able to adapt these features to the target domain (in this case, rose leaves) using a smaller labeled dataset, and generally improves sample efficiency compared to training from scratch [19].

The remaining part of the article describes the model architectures that were evaluated and the end-to-end training workflow, and then presents the interpretation of the empirical results, including figures from both preprocessing variants. Rather than proposing methodological innovations, the work integrates established techniques into a coherent, cross-disciplinary pipeline consisting of a reproducible comparison of training from scratch versus transfer learning using lightweight training on a rose leaf dataset, a practical leaf isolation preprocessing method that is evaluated against a grayscale baseline, and a deployment-oriented packaging of trained models (including preprocessing metadata and ONNX export) so that the inference can replicate the training assumptions accurately.

## 2. Architecture of the Decisional Pipeline

In the context of agricultural use cases, the challenges of pattern recognition are different than those found in industry, where recognition is based on features that do not change, like area, perimeter, or image moments. Statistically oriented models, primarily deep learning, provide an advantage for the agricultural domain due to the variability of biological data. Deep learning is used to automatically extract meaningful information from plant images that allows for robust disease identification regardless of how much the environmental conditions differ, how variable the shape of leaves is, or how variable the manifestation of diseases is.

An end-to-end pipeline was proposed for automated rose leaf disease detection, providing the opportunity for the integration of machine vision and robotics through the collaboration of all key stages of the decision-making process:

1. Data acquisition: Autonomous robotic platforms, including trolleys, robotic arms, or stationary units, have been equipped with cameras to collect systematic images of rose crops. These robots will traverse greenhouse paths, position the camera(s) for optimal leaf imaging, and continue to operate autonomously so that there is a continuous supply of images for the analysis, thereby providing the capability to perform high-throughput and consistent monitoring.
2. Preprocessing: Raw images acquired by either robotic or manually operated systems are converted into a standardized format using techniques such as HSV-based leaf masking or grayscale conversion. The preprocessing steps remove background noise and create standardized images that allow for increased reliability of the subsequent classification. This step is critical for increasing the robustness of the model, particularly in environments with varying levels of lighting and cluttered backgrounds.
3. Model inference: The CNN model is at the center of the pipeline and has been trained to differentiate between healthy and diseased leaves. The CNN model uses the preprocessed images as input and generates a classification output (healthy or diseased).
4. Decision and robotic action: Depending on the output generated by the model, the system can generate a variety of responses [20]. For example, a robot could separate affected plants, apply treatment to affected areas, or identify areas for human review. Therefore, the decision-making process is closed loop from perception (machine vision) to action (robotics) and supports a complete automated disease management process. Additionally, all results are documented along with metadata (location, cultivar, and lighting) and support traceability and continuous improvements.
5. Monitoring and retraining: Continuous post-deployment monitoring is necessary to determine if there has been a shift in the underlying data distribution (dataset drift) and to ensure that the model does not silently degrade (due to changes in lighting, seasonal variations, etc.). Changes in the environment, new cultivars, new disease stages, etc., are common sources of hidden technical debt in DL systems [21], and should be continually monitored and addressed through periodic relabeling and retraining.

Although the entire system was developed to functionally support robotic platforms and automate end-to-end, this paper focuses on the decisional core of the pipeline, specifically steps 2 (preprocessing) and 3 (model inference), which make up the intelligent core of the pipeline, converting raw images into actionable knowledge. Through the rigorous design and evaluation of these two stages, the work provides a reproducible framework for robust, automated disease detection, which can be easily integrated with upstream (robotic image acquisition) and downstream (robotic intervention) systems.

To evaluate the performance of the two model families for the binary (healthy/sick) classification task, we selected a collection of models ranging from a simple baseline model to deep transfer learning architectures. We selected these model families to address practical concerns that arise in a small-data setting, with ResNet being a good fit in this case because of its residual connections, which enable a proven, compact architecture to learn robust visual disease cues from a real-world dataset while also taking advantage of its pretrained features, keeping training stable and comparisons reproducible. First, it is beneficial to select a computationally inexpensive baseline model (SimpleCNN): if the smaller model performs well, it is likely to be simpler to implement on limited hardware and serves as a reference point for whether additional complexity is justified. Second, moderate-sized backbones (ResNet-18) are commonly selected when the available amount of labeled data may not be sufficient, the reason being that pretrained features typically enhance generalization and reduce the amount of time needed for training. Third, selecting a higher-capacity backbone (ResNet-50) provides an opportunity to test whether increased depth and representational power result in measurable gains, or simply increase the likelihood of overfitting and computational costs. The total set of models spans a controlled and interpretable spectrum of capacity and compute, allowing for a direct comparison of the same preprocessing and training pipeline.

### 2.1. SimpleCNN

A minimal convolutional baseline for computational efficiency, SimpleCNN is trained from scratch, so it must learn both low-level features (edges and textures) and task-specific disease cues from the rose dataset alone.

**Figure 1:**
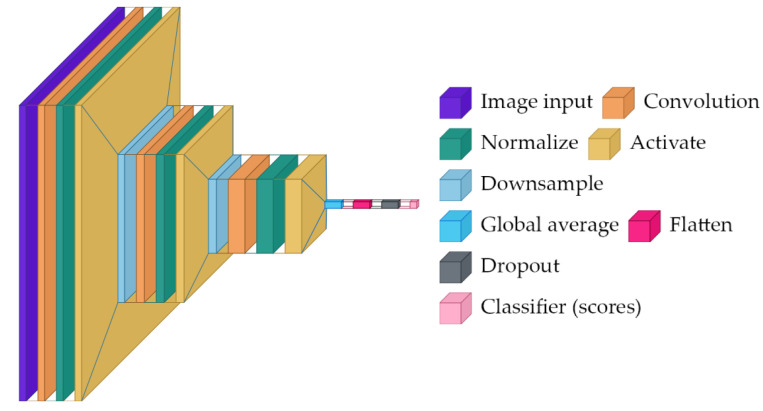
High-level architecture diagram for SimpleCNN, showing repeated convolutional feature extraction, pooling, and a small final classifier.

The architecture presented in Figure 1 is comprised of three sequential convolutional blocks:

- Block 1: 3 → 32 channel convolution (3 × 3 kernel, padding = 1), batch normalization, ReLU activation, and 2 × 2 max pooling.
- Block 2: 32 → 64 channel convolution (3 × 3 kernel, padding = 1), batch normalization, ReLU activation, and 2 × 2 max pooling.
- Block 3: 64 → 128 channel convolution (3 × 3 kernel, padding = 1), batch normalization, ReLU activation, and adaptive average pooling to 1 × 1 spatial dimensions.
- Classifier: flattened features, 20% dropout, and linear layer mapping 128 features to 2 class logits.

This model serves as a reference point—it is fast and small but may struggle if the disease cues are subtle and the dataset is limited.

### 2.2. ResNet-18

ResNet-18 is a residual network with 18 layers pretrained on ImageNet (1.4 M images, 1000 classes). In this project, it is fine-tuned for a two-class task by replacing the final classifier layer and then continuing training on the rose dataset.

The key concept is the residual (skip) connection: instead of forcing a stack of layers to learn a complete transformation of its input, a residual block learns a correction on top of the input. In simplified form, if the input to a block is x and the block computes F(x), the output is x + F(x). This structure improves optimization and helps gradients propagate through deep networks [14].

**Figure 2:**
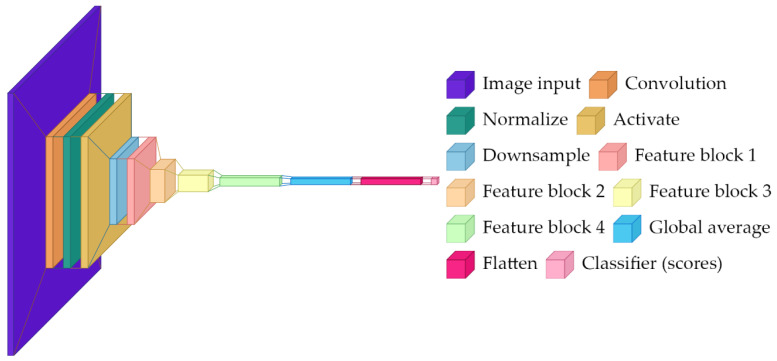
High-level architecture diagram for ResNet-18.

The network presented in Figure 2 contains the following:

- Initial 7 × 7 convolution (64 channels) followed by batch normalization, ReLU, and 3 × 3 max pooling.
- Four residual stages with {2, 2, 2, 2} basic residual blocks at {64, 128, 256, 512} channels.
- Each basic block contains two 3 × 3 convolutions with batch normalization and a skip connection.
- Global average pooling reducing spatial dimensions to 1 × 1.
- Replaced classification head: original 1000-way classifier removed, new linear layer maps 512 features to 2 class logits.

Global average pooling is important for interpretability: rather than relying on a fixed location in the image, it summarizes each learned feature across the whole spatial field. The final linear layer then combines these features to produce logits for the two classes. All convolutional layers remain trainable to allow full fine-tuning, but because the model starts from pretrained weights, it can converge faster and often generalize better than a same-sized network trained from scratch.

### 2.3. ResNet-50

ResNet-50 is a deeper residual network with 50 layers, also pretrained on ImageNet. Compared to ResNet-18, this architecture uses bottleneck residual blocks for greater representational capacity. Intuitively, it can represent more complex functions and potentially separate more subtle visual differences, but it also has more parameters and may overfit more easily when data are limited.

The defining element is the bottleneck block, which uses 1 × 1 convolutions to temporarily reduce the channel dimension, applies a 3 × 3 convolution at that reduced dimension, and then expands back. The 1 × 1 convolutions act like learned channel mixers: they combine information across channels without looking at a larger spatial neighborhood.

**Figure 3:**
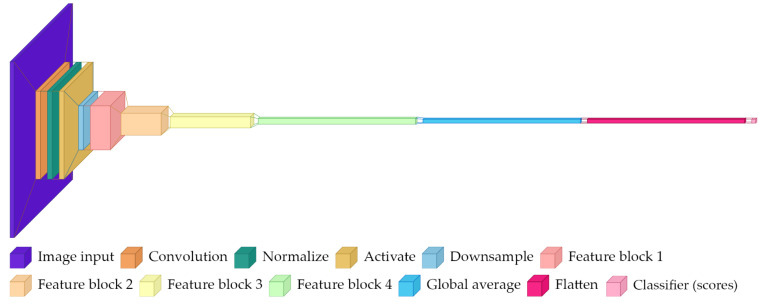
High-level architecture diagram for ResNet-50.

The architecture presented in Figure 3 includes the following:

- Identical initial convolution and pooling to ResNet-18.
- Four residual stages with {3, 4, 6, 3} bottleneck blocks at base channels {64, 128, 256, 512}.
- Each bottleneck block uses 1 × 1 convolutions for dimensionality reduction/expansion around a 3 × 3 convolution.
- Final feature dimension of 2048 channels after global average pooling.
- Replaced classification head: new linear layer maps 2048 features to 2 class logits.

This model tests whether added capacity improves performance on the available data, and whether early stopping and augmentation are sufficient to prevent overfitting in a small dataset regime.

## 3. Implementation

The goal of this section is to clearly document the end-to-end process to train and test the rose leaf disease classifier, emphasizing both reproducibility and transparency of design decisions (particularly those regarding preprocessing), to a multidisciplinary audience. A standard directory-based dataset interface is used to load images and associate them with a class label, based on their parent class folder name. Using a group-aware 5-fold cross-validation, generated through a random seed, directly comparable results are created, which reduce the sensitivity to any one particular data partition. Additionally, grouping is used to prevent near-duplicate images from being split across folds, which is essential in avoiding leakage and yields a more reliable estimate of generalization. In this work, a group is defined as a cluster of near-duplicate images identified through visual similarity analysis, without systematical grouping of images belonging to the same plant or acquisition session.

Four versions of preprocessing alternatives were tested to determine how much preprocessing would affect the performance of detecting diseases:

- Original image: the complete information of the image is kept.
- Grayscale: images are converted to grayscale (repeated to three color channels to be compatible with ImageNet-trained backbones).
- Leaf mask RGB: the leaf is masked using a conservative HSV-based method, after which standard augmentation is performed. RGB color information is retained.
- Leaf mask grayscale: both transformations are performed.

Each alternative version was tested independently using separate cached tensors and separately saved model checkpoints. As mentioned earlier, leaf images collected in natural environments have several issues that detract from the overall quality of the images, including the presence of distracting structural elements in the background (pots, soil, etc.), as well as significant variations in illumination. In addition, there is a relatively small amount of training data available to the CNN. As a result, the CNN may partially “learn” spurious correlations from these types of backgrounds (e.g., a particular type of bench or lighting condition that occurs more frequently in a single class). Therefore, the motivation behind leaf masking is two-fold: reduce the amount of background noise that is present in the image so that the model is forced to rely on the leaf texture and lesion characteristics instead of the surrounding environment; also, simplify and stabilize the application of random augmentation so that random crops and rotations of the image are more likely to contain informative aspects of the leaves.

This is not a complete segmentation system but rather a lightweight, conservative preprocessing stage intended to provide safe operation across a variety of input images.

The leaf-masking stage was implemented in terms of a simple HSV thresholding and cropping operation: First, convert the input image to RGB and then to HSV, then identify “leaf-like” pixels by thresholding the HSV values (a roughly green hue interval with minimal saturation and value constraints). Afterwards, apply simple mask cleaning filters (median filter followed by max filter) to eliminate small holes and join adjacent leaf regions together. If no valid mask is obtained, fall back to the original image (safe behavior), otherwise calculate the mask bounding box, add a small padding factor to it, crop to that area, and set all non-leaf pixels to zero.

In order to keep the number of scene variables low while also being cheap to compute and robust enough to serve as a default in a small-data setting, the above approach is deliberately kept simple. All images are rescaled to 224 × 224 inputs to meet the expected size of common ImageNet-pretrained models. Stochastic augmentation dominated by aspect-preserving random resized crops (scale 0.8–1.0, aspect ratio 0.9–1.1), horizontal flip, small rotation (±10 degrees), and slight photometric perturbation (e.g., brightness/contrast jitter, sharpness adjustment, blur, and histogram equalization) is used during training. Deterministic resize and center cropping are used for validation and testing in order to provide consistent and comparable assessments. Moreover, these steps are used to further augment the dataset by enriching the number of images in the healthy class to reach a balanced training dataset.

Transfer learning backbones use either the mean and standard deviation for normalization, as defined in the weights used for the pretraining where possible, otherwise standard ImageNet normalization is used. It is essential that the normalization parameters and the configuration of the preprocessing for a specific experiment are saved along with each model checkpoint so that the assumptions made at training time can be replicated during inference.

Training iterates through a list of architectures (ResNet-50, ResNet-18, and SimpleCNN) and uses Adam with a learning rate of 3 × 10^−5^ and cross-entropy loss for optimization. To mitigate the problem of overfitting due to limited amounts of data, early stopping and checkpoint selection are performed with the patience of five epochs to monitor whether validation loss is improving.

A cross-entropy loss function is being used because the model produces probability distributions for the two classes, and the loss function will punish wrong confident predictions more than wrong predictions with uncertainty. Adam is a type of optimizer that uses the running average of the gradient to dynamically adjust the step size for each parameter and, therefore, is a typical choice for fine-tuning. Early stopping is monitoring validation loss instead of training loss; when validation loss ceases to improve, it is a practical indication that further training will only improve performance on the training set and not improve generalization.

The model checkpoint includes the learned weights, the class labels, the normalization parameters, the identifier for the architecture, and the configuration of the preprocessing used for that particular experiment. This allows the ability to switch from one experiment to another at inference time by loading and then applying the correct preprocessing pipeline for each respective checkpoint.

For deployment, the trained neural network can be exported to the Open Neural Network Exchange (ONNX) format [22]. The ONNX export captures both the model computation graph and weights, allowing cross-platform inference to be executed in a target environment. As a reminder, the ONNX export generally does not capture the entire preprocessing pipeline of the original images (resized/cropped, optionally masked leaves, and normalized). Therefore, the deployment of the trained model should recreate the same preprocessing configuration saved with the checkpoint (resized/cropped policy, optional leaf masking, and normalization parameters). In practice, ONNX Runtime (ORT) has a high-performance inference engine that can execute ONNX models on CPU or GPU [23]. When deployed to a production environment with a CUDA-capable GPU, ORT can be configured to use GPU execution to increase throughput and reduce latency, which is critical when processing many plants per hour.

## 4. Experimental Results

Experiments have been carried out on a dataset of 318 real-world images of roses, which were manually acquired under unconstrained conditions using consumer devices, such as smartphones and digital cameras. The images are grouped into two labeled classes: 89 images of healthy roses and 229 images of sick roses affected by powdery mildew. Given their unconstrained nature, the inputs exhibit variation in viewpoint, distance, illumination, background elements, and camera processing, providing a realistic starting point for evaluating disease classification performance in an agricultural environment.

Moreover, near-duplicate images were identified using a 64-bit perceptual difference hash (dHash). Images were converted to grayscale, resized, and hashed based on horizontal pixel gradients. Pairwise Hamming distances between hashes were computed within each class, and images with distance ≤ 6 were grouped using a union-find clustering scheme, which led to the finding of 14 duplicate clusters, as highlighted in Table 1. The resulting groups were used in the preparation of five group-aware cross-validation splits, so that the duplicated images would not spill in different splits.

All experiments were conducted using Python 3.10.12 on a Linux environment (kernel 6.6.87.2, glibc 2.35) running under WSL2. The software stack consisted of PyTorch 2.9.1 + cu128, Torchvision 0.24.1 + cu128, NumPy 2.2.6, Pandas 2.3.3, scikit-learn 1.7.2, and SciPy 1.15.3. The hardware on which these were executed on consisted of an NVIDIA GeForce RTX 4070 (Nvidia Corporation, Santa Clara, CA, USA) Laptop GPU [24] using 4608 CUDA cores with CUDA 12.8, with the system reporting CUDA as the active device, an Intel Core i9-14900HX processor (Intel Corporation, Santa Clara, CA, USA), and 32 GB RAM. This configuration ensured GPU-accelerated training and inference for all deep learning components.

We report both validation and held-out test metrics across three model architectures. To assess preprocessing sensitivity, results are reported for two variants: with and without leaf cropping.

### 4.1. Preprocessing Visualization

**Figure 4:**
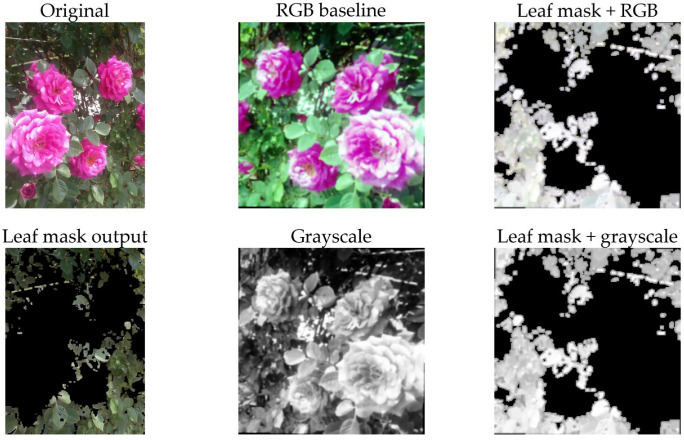
Examples of the four preprocessing variants on an image of healthy rose plant. (**Top row**): original image (**left**), RGB baseline (**middle**) and HSV-based leaf mask output (**right**). (**Bottom row**): HSV-based leaf mask output (**left**), grayscale version of the processed image (**middle**) and grayscale version of the processed leaf mask (**right**).

**Figure 5:**
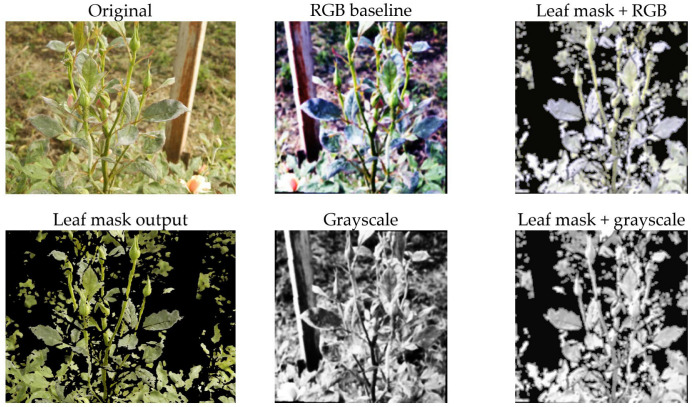
Examples of the four preprocessing variants on an image of rose plant afflicted by powdery mildew. (**Top row**): original image (**left**), RGB baseline (**middle**) and HSV-based leaf mask output (**right**). (**Bottom row**): HSV-based leaf mask output (**left**), grayscale version of the processed image (**middle**) and grayscale version of the processed leaf mask (**right**).

Beginning with the images in Figure 4 and Figure 5, the four preprocessing approaches shown were tested in the current work. The top row of the 2 × 3 matrix is an image prior to processing (left), followed by the RGB baseline (middle), which has undergone the core preprocessing steps, and the HSV-based leaf mask output (right). The bottom row displays an example of the HSV-based leaf mask output (left), the grayscale version of the processed image (middle), and an example of the grayscale version of the processed leaf mask (right). All versions of the image underwent the same downstream augmentation scheme during the training process. The purpose of this graph was to provide a visual representation of the differences in preprocessing.

From the graph, there are two visually evident results. First, the leaf mask, as illustrated in Figure 4 and Figure 5, is not performing any form of pixel perfect segmentation. Instead, it is providing a conservative crop-and-clean-up function. The function is designed to keep the leaf area clean and suppress all other areas that appear to be part of the background. This reduces the probability of the classifier learning shortcut methods from the pots, soil, and/or greenhouse structures. Second, the grayscale conversion removes all color-based cues from the image, requiring the model to rely on the texture and intensity characteristics of the leaf (such as speckling, blotches, and vein disruption). If the performance of the model remains consistent when using grayscale versus RGB images, then this would be evidence that the model is not relying on color variations as the primary method to identify the leaves. Conversely, if the model performs poorly when using grayscale images, this would suggest that the model has been trained to use the color variations found in the leaf images.

### 4.2. Ablation Study (Hyperparameters and Data Augmentation)

**Figure 6:**
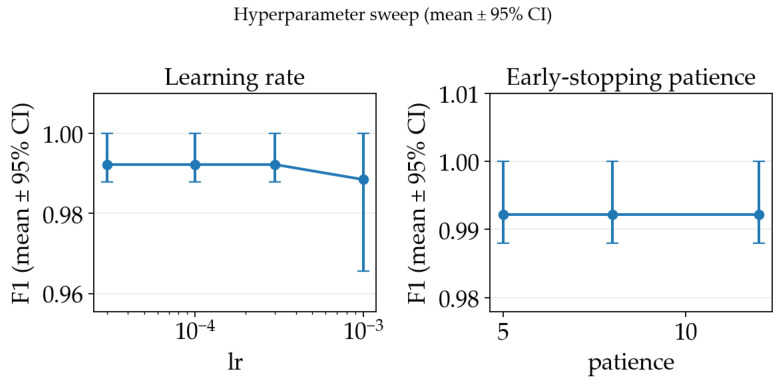
Hyperparameter ablation study mean F1-score across splits together with 95% bootstrap confidence intervals.

In order to select appropriate hyperparameters for the training process, we have conducted an ablation study on the training pipeline using the RGB baseline configuration using a ResNet18 classifier and a 3-fold cross-validation subset. For each of the settings’ configurations, we report the mean F1-score across splits together with 95% bootstrap confidence intervals (CIs) computed over the split-level scores. Firstly, we swept the Adam learning rate and early stopping patience, which are the key training hyperparameters used by our training loop. As illustrated in Figure 6, the sweep exhibited a broad plateau of near-identical performance for learning rates in the $\left[3\times 10^{-5},3\times 10^{-4}\right]$ range and patience values between 5 and 12 epochs, while a higher learning rate ($10^{-3}$) consistently reduced performance. Based on mean F1-score, we selected $lr=3\times 10^{-5}$ and patience=5 as the best-performing and simplest among the top-performing hyperparameter choice.

**Figure 7:**
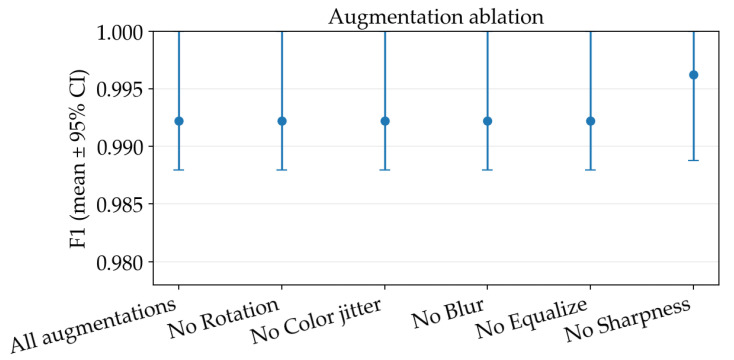
Augmentation steps ablation study mean F1-score across splits together with 95% bootstrap confidence intervals.

Using the resulted hyperparameters, we then performed an additional study on individual augmentation components from the base recipe: rotation, color jitter, Gaussian blur, histogram equalization, and sharpness adjustment, to quantify their marginal contribution. Removing most of them produced no measurable change relative to the base configuration (mean accuracy and F1 remained unchanged within the resolution of our three-split evaluation and with fully overlapping CIs), with the exception of removing the random sharpness adjustment yielded a small but consistent improvement, increasing accuracy to 0.9947 (95% CI $\left[0.9841,1.0000\right]$) and F1 to 0.9963 (95% CI $\left[0.9888,1.0000\right]$), as observed in Figure 7.

The ablation study was conducted on a representative ResNet-18 + RGB configuration with 3-fold cross-validation to limit computational costs. Given the observed low sensitivity of performance to hyperparameter variations, the selected settings were reused across all architectures for consistency. While this unified setup supports comparability, it does not exclude the possibility that architecture-specific tuning could yield marginal gains.

### 4.3. Training Dynamics

**Figure 8:**
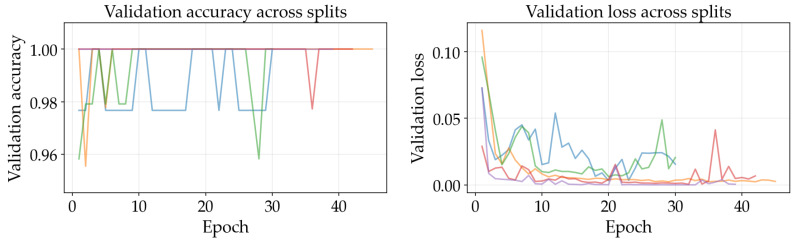
Training and validation curves for ResNet-50 with leaf mask RGB preprocessing.

**Figure 9:**
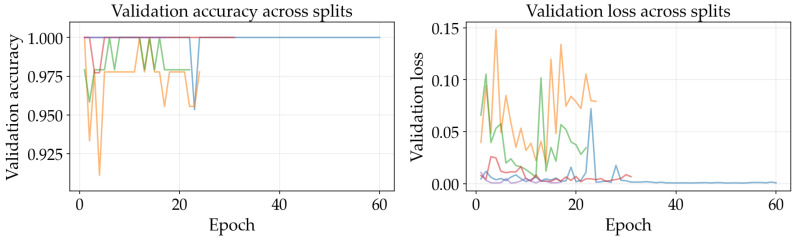
Training and validation curves for ResNet-18 with leaf mask RGB preprocessing.

**Figure 10:**
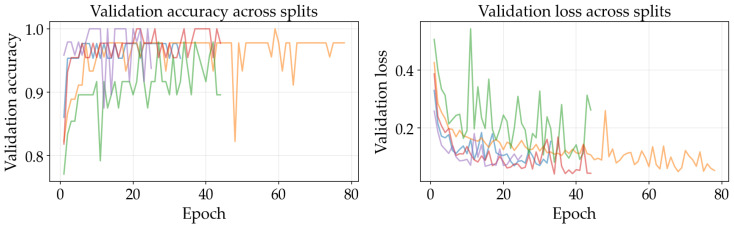
Training and validation curves for SimpleCNN with leaf mask RGB preprocessing.

**Figure 11:**
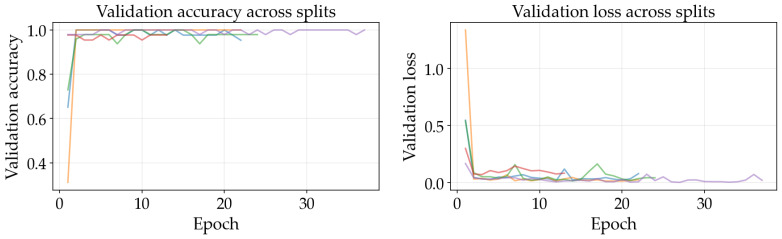
Training and validation curves for ResNet-50 with grayscale preprocessing.

**Figure 12:**
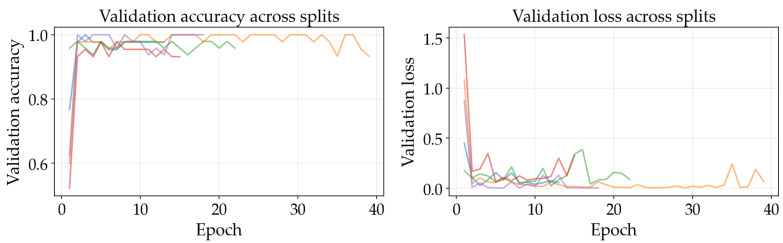
Training and validation curves for ResNet-18 with grayscale preprocessing.

**Figure 13:**
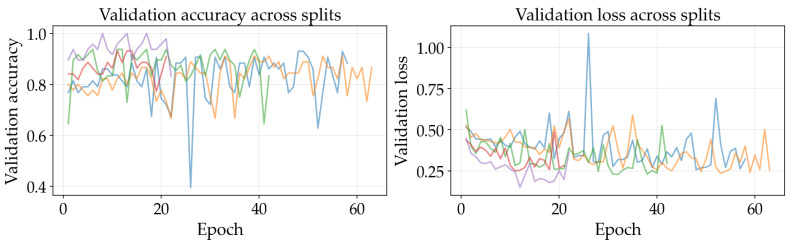
Training and validation curves for SimpleCNN with grayscale preprocessing.

The results illustrated in Figure 8, Figure 9, Figure 10, Figure 11, Figure 12 and Figure 13 illustrate validation performance curves for each of the four models used across the leaf mask RGB and grayscale preprocessing options since they capture the variability of the system. The two metrics plotted against the epoch number are as follows: (i) cross-entropy loss, which is a measure of how accurately the model’s prediction matches the ground truth label, and (ii) accuracy, which is the ratio of correct predictions. Because loss typically tracks the earliest sensitivity during the training phase, it is often considered the most informative metric until the accuracy saturates. All metrics are reported across training splits.

All of the ResNet models demonstrated rapid convergence. This is consistent with the nature of transfer learning, where a pretrained network begins from an existing representation of general visual features; therefore, the primary requirement is to adapt the existing representation to be able to distinguish the two classes within the provided dataset (see Figure 8, Figure 9, Figure 10 and Figure 11). Conversely, the lightweight SimpleCNN learned to represent the visual features from the beginning and had a much slower increase in both loss and accuracy (see Figure 10 and Figure 13).

In Figure 8, Figure 9, Figure 10, Figure 11, Figure 12 and Figure 13 each color represents one of the five folds used for cross-validation consistently across representations. The validation curves shown in Figure 8, Figure 9 and Figure 10 exhibit validation loss oscillations, which are particularly visible for the higher-capacity ResNet-50 model trained on the leaf-masked RGB data. This behavior can be attributed to the small-data regime with stochastic data augmentation and a limited validation split, considering that validation loss can fluctuate even when overall accuracy boundaries remain stable. Since higher-capacity models such as ResNet-50 can adapt rapidly to individual samples, this produces minor shifts in prediction confidence and noticeable oscillations of loss, even after accuracy has saturated near its maximum and no true performance degradation is seen. These conditions further validate the decision to select checkpoints based on the minimum validation loss in contrast to maximum accuracy.

Generally speaking, training–validation differences were minimal in these experiments. However, since the validation set was small in size, it is possible that a high validation accuracy may be reported for a particular model while still having uncertainty regarding its ability to generalize to other test conditions. As such, early stopping may provide some level of mitigation of this concern by allowing selection of the model checkpoint corresponding to the minimum validation loss and ceasing model training after a predetermined “patience” window (if no improvement is noted).

Comparing the results of using leaf masks in RGB format versus converting the images to grayscale, the resulting performance curves are essentially equivalent. Therefore, there appears to be no evidence that the models are required to have strong reliance on whether the color is maintained or whether the initial preprocessing steps are applied in a lightweight manner to isolate the leaf before they can effectively discriminate between images representing healthy vs. sick leaves. On the other hand, leaf masking is likely to remain a reasonable approach to address variations in background and illumination that may occur outside of what is represented in the current training data.

### 4.4. Test Set Performance

**Figure 14:**
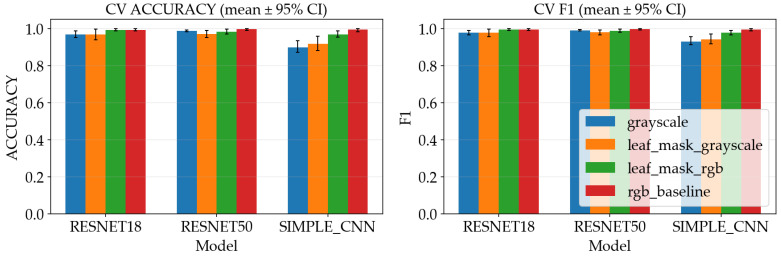
Metrics comparison across models and preprocessing variants.

Table 2 and Figure 14 summarize the metrics resulting from the aggregated out-of-fold predictions across five CV folds. The test set is intended to approximate performance on new images that were not used to tune the model parameters. However, because the overall dataset is small, a single misclassification can meaningfully change the reported percentage, so the numbers should be treated as an estimate rather than a guarantee.

The table shows that both ResNet models reach the highest test accuracy under all preprocessing variants, while SimpleCNN is slightly lower. This is consistent with the training dynamics: transfer learning makes it easier to learn useful image features from limited data, whereas a small model trained from scratch may underfit subtle disease cues or may require more data to reach the same level of generalization.

The grouped bar chart makes the comparison easier to observe, where performance differences between preprocessing variants are small relative to the differences between model families. In practical terms, this suggests that for this dataset the augmentation and normalization pipeline already provides some robustness, and that either preprocessing variant could be used without a large accuracy penalty. That said, deployment conditions often differ from training conditions—leaf masking may still be beneficial when backgrounds are more cluttered or when the camera setup varies.

Table 3 places the present work in the context of prior studies. Compared to large-scale datasets such as Ferentinos [13], which evaluates multi-class classification across 58 categories using tens of thousands of images, the current study focuses on a small, real-world dataset with unconstrained acquisition conditions. In contrast to prior works that typically rely on single training/test splits [13,15], we employ group-aware cross-validation to reduce leakage and better estimate generalization. Therefore, while the reported performance is numerically comparable, the evaluation setting is more constrained and reflects a more realistic deployment scenario.

**Figure 15:**
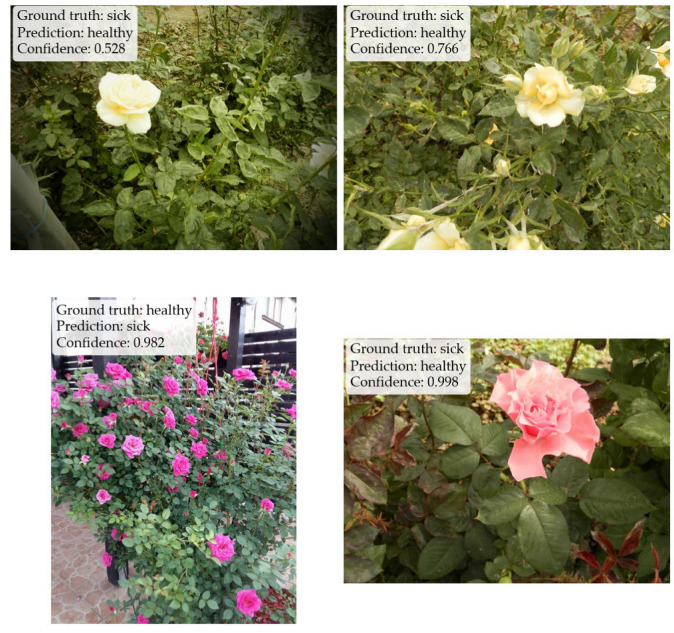
Four examples of misclassified images, labeled with the ground truth, the predicted label, and the confidence score.

**Figure 16:**
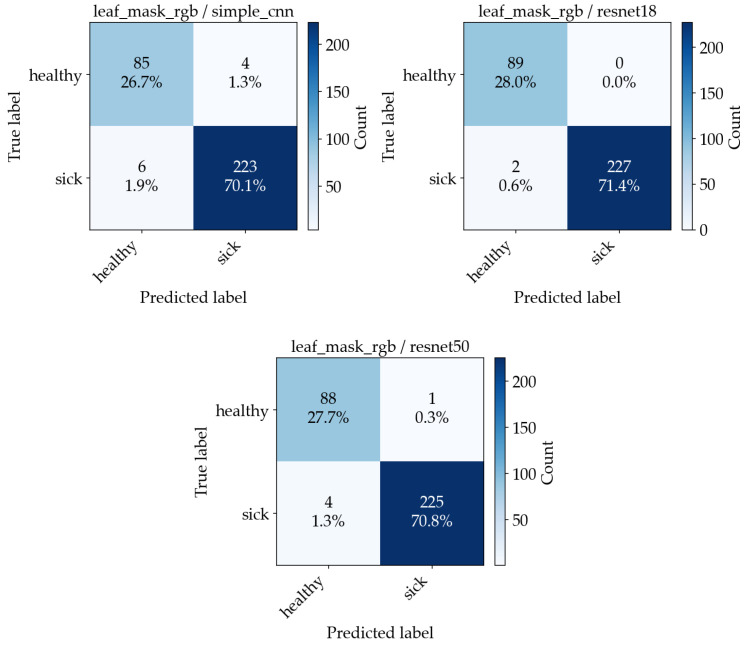
Confusion matrices from the aggregated out-of-fold predictions across 5 CV folds for the leaf mask RGB experiment ((**Left**)—SimpleCNN, (**Middle**)—ResNet18, (**Right**)—ResNet50).

**Figure 17:**
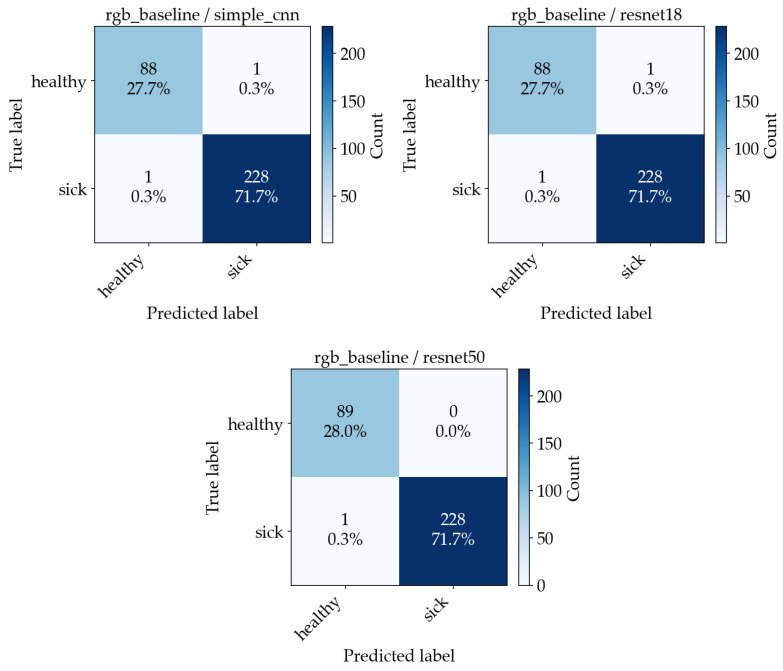
Confusion matrices from the aggregated out-of-fold predictions across 5 CV folds for the RGB baseline experiment ((**Left**)—SimpleCNN, (**Middle**)—ResNet18, (**Right**)—ResNet50).

Figure 15 shows four examples of misclassified images, along with their predicted and expected outputs. Accuracy alone does not distinguish between false negatives and false positives. To make the error types explicit, confusion matrices are computed. Figure 16 and Figure 17 show the resulting confusion matrices for the two best-performing preprocessing variants (leaf mask RGB and RGB baseline), with one matrix per model. Each matrix counts how often a true class (row) is predicted as each class (column). Values concentrated on the diagonal indicate correct classification, while off-diagonal cells correspond to specific error types (healthy predicted as sick, or sick predicted as healthy). 

Moreover, we have measured the inference and training time to evaluate real-world performance. As seen in Table 4, across all preprocessing variants, inference on GPU is sub-millisecond to ~2 ms per image (≈500–2000 images/s), so the deployment time cost is low. ResNet-50 is consistently slower than ResNet-18 (roughly 2–3× higher inference latency and ~2–4× longer training time per split), while SimpleCNN is generally closer to ResNet-18 for inference but can take longer to train due to requiring more epochs before early stopping.

## 5. Conclusions

Several key points about model performance and the potential application of the rose leaf disease classifier can be derived from the analysis of the evaluation. In addition to good performance on the binary disease classification problem by all three architecture designs, the fact that the ResNet models are able to achieve very high validation accuracy so rapidly using pretrained weights from ImageNet is certainly positive news for the use of deep learning in automated leaf screening. Further, the fact that SimpleCNN’s performance grows gradually through the training process is consistent with the expectation that SimpleCNN needs to learn feature representations from scratch, whereas ResNet will inherit many of these representations from the pretraining on ImageNet.

Both preprocessing methods (leaf mask RGB vs. grayscale) were shown to produce virtually equivalent results, indicating that the architectures being evaluated are relatively insensitive to the specific preprocessing method used. Since the leaf segmentation step may not necessarily be required for accurate disease detection on this particular dataset, this makes deploying the leaf disease classifier easier.

The high accuracy of the learned decision boundary suggests that it has generalized reasonably well to the held-out test split; however, since the size of the dataset is relatively small, there could be some combination of statistical uncertainty, near duplication of images within the training set, or some form of split leakage effect contributing to the perfect scores obtained. Therefore, care should be taken in interpreting very high accuracy values, and some of the implementation decisions made (such as augmentation, converting grayscale to three channels, caching, and class balancing) were made to mitigate potential issues.

Additional image data collected at a wider variety of light levels and/or backgrounds with various disease severities would provide a much better evaluation of model robustness and allow for a more meaningful comparison of the performance of different architectures under the same conditions. Also, developing a version of the classifier capable of multi-class disease identification (i.e., identifying among powdery mildew, black spot, and rust, etc.) would greatly increase its applicability to growers. Additionally, incorporating stronger regularization schemes (and systematically experimenting with other forms of regularization, such as weight decay, learning rate scheduling, and freezing layers) would likely result in less overfitting to the training set, particularly when evaluating performance on small datasets. As an added benefit, the modular nature of the training registry allows for easy integration of additional backends to support future enhancements. Lastly, packaging the trained models into ONNX format and then serving them using ONNX Runtime (on either the CPU or GPU) would make deployment a much simpler task and ensure that the preprocessing steps are consistent with the trained experiment configurations [22,23].

## Figures and Tables

**Table 1:** Near-duplicate analysis results.

Label	Number of Groups	Size of Group
Sick	2	4
Sick	2	3
Sick	9	2
Healthy	1	2

**Table 2:** Model test metrics resulting from experiments.

Model	Preprocessing Method	Precision Mean	Recall Mean	F1 Mean	AUC Mean
Grayscale	ResNet-18	0.987 ± 0.0152	0.9704 ± 0.0257	0.9782 ± 0.0127	0.9975 ± 0.0032
Grayscale	ResNet-50	0.9957 ± 0.0065	0.9868 ± 0.0093	0.9911 ± 0.0036	0.998 ± 0.0027
Grayscale	SimpleCNN	0.9155 ± 0.033	0.9485 ± 0.0251	0.9311 ± 0.0217	0.9487 ± 0.0244
Leaf-masked RGB	ResNet-18	1.0 ± 0.0	0.9912 ± 0.0088	0.9956 ± 0.0045	1.0 ± 0.0
Leaf-masked RGB	ResNet-50	0.9956 ± 0.0067	0.9824 ± 0.0131	0.9889 ± 0.0089	0.9995 ± 0.0007
Leaf-masked RGB	SimpleCNN	0.9836 ± 0.0122	0.9742 ± 0.0221	0.9786 ± 0.0119	0.9944 ± 0.0048
Leaf-masked grayscale	ResNet-18	0.9816 ± 0.0139	0.9736 ± 0.0265	0.9775 ± 0.0201	0.9909 ± 0.0122
Leaf-masked grayscale	ResNet-50	0.9868 ± 0.0155	0.9742 ± 0.0221	0.9802 ± 0.0137	0.9941 ± 0.0089
Leaf-masked grayscale	SimpleCNN	0.9434 ± 0.0357	0.9428 ± 0.0439	0.9417 ± 0.0265	0.9754 ± 0.0163
RGB	ResNet-18	0.9952 ± 0.0071	0.9956 ± 0.0067	0.9953 ± 0.0047	1.0 ± 0.0
RGB	ResNet-50	1.0 ± 0.0	0.9956 ± 0.0067	0.9978 ± 0.0034	0.9993 ± 0.0011
RGB	SimpleCNN	0.9962 ± 0.0058	0.9962 ± 0.0058	0.9962 ± 0.0058	0.9993 ± 0.001

**Table 3:** Comparison with prior work.

Study	Dataset Size	# Classes	Task Type	Evaluation Protocol	Reported Performance
Ferentinos [13]	87,848 images	58 classes	Multi-crop, multi-disease classification	Training/test split	99.53% accuracy
Kaur et al. [15]	PlantVillage dataset (large-scale)	4 classes (grape diseases)	Multi-class classification	Training/test split	98.7% accuracy, 0.97 F1 Score
This work	318 images	2 classes	Binary classification (rose)	5-fold group-aware CV + test set	Up to ~0.99 F1/AUC

**Table 4:** Running time resulting from experiments.

Model	Preprocessing Method	Inference Time (ms)	Training Time (ms)
Grayscale	ResNet-18	0.519	29.114
Grayscale	ResNet-50	1.755	86.319
Grayscale	SimpleCNN	0.602	47.114
Leaf-masked RGB	ResNet-18	0.508	37.81
Leaf-masked RGB	ResNet-50	1.724	129.903
Leaf-masked RGB	SimpleCNN	0.591	47.126
Leaf-masked grayscale	ResNet-18	0.528	25.905
Leaf-masked grayscale	ResNet-50	1.705	94.319
Leaf-masked grayscale	SimpleCNN	0.609	55.936
RGB	ResNet-18	0.692	36.457
RGB	ResNet-50	1.994	116.569
RGB	SimpleCNN	0.724	70.648

## Data Availability

The data that support the findings of this study are available upon request from the corresponding author.

## Abbreviations

The following abbreviations are used in this manuscript:

CNN	Convolutional Neural Network
DL	Deep Learning
ResNet	Residual Network
ReLU	Rectified Linear Unit
RGB	Red, Green, Blue
HSV	Hue, Saturation, and Value
ONNX	Open Neural Network Exchange
CV	Cross-Validation
